# Plate-like Alginate Microparticles with Disulfiram–SPIO–Coencapsulation: An In Vivo Study for Combined Therapy on Ovarian Cancer

**DOI:** 10.3390/pharmaceutics13091348

**Published:** 2021-08-27

**Authors:** Meng-Yi Bai, Mu-Hsien Yu, Ting-Teng Wang, Shiu-Hsin Chen, Yu-Chi Wang

**Affiliations:** 1 Graduate Institute of Biomedical Engineering, National Taiwan University of Science and Technology, TR-917, AAEON Building, No.43, Keelung Rd., Sec.4, Da’an Dist., Taipei 10607, Taiwan; mybai@mail.ntust.edu.tw (M.-Y.B.); stanley777100@gmail.com (T.-T.W.); M10623202@mail.ntust.edu.tw (S.-H.C.); 2 Biomedical Engineering Program, Graduate Institute of Applied Science and Technology, National Taiwan University of Science and Technolo-gy, TR-917, AAEON Building, No.43, Keelung Rd., Sec.4, Da’an Dist., Taipei 10607, Taiwan; 3 Adjunct Appointment to the Department of Biomedical Engineering, National Defense Medical Center, Taipei 11490, Taiwan; 4 Department of Obstetric and Gynecology, Tri-Service General Hospital, National Defense Medical Center, Taipei 11490, Taiwan; hsienhui@ms15.hinet.net

**Keywords:** electrospray, natural polymer, disulfiram, alginate, biomaterials

## Abstract

Disulfiram is a drug used to support the treatment of chronic alcoholism. Recently, it has been found to have an off-label ability to inhibit the growth of ovarian cancer cells. However, the original formulation was designed for use via oral administration, which is not suitable to be given by a direct spray on the affected area. Therefore, in this study, we designed and prepared alginate (ALG) microparticles loaded with disulfiram and superparamagnetic iron oxide (cross-linking disulfiram/SPIO/ALG MPs), which have great potential application for inhibiting the growth of ovarian cancer simultaneously via two treatments, i.e., chemotherapy and hyperthermia. The drug-encapsulating alginate microparticles were prepared using an electrospray system and then cross-linked with calcium chloride ions. The particles were observed by optical microscopy and scanning electron microscopy, and found to be approximately 200 μm in diameter. The disc-shape morphology of the microparticles could be controlled by up to 95%. The drug-encapsulation efficiency of the microparticles reached 98%, and the suppression of tumor growth for the free-form disulfiram-treated group and disulfiram/SPIO/ALG MPs-treated group were 48.2% and 55.9% of tumor volume reduction, respectively, compared with a cisplatin-treated group. A hyperthermic effect can be achieved by applying a magnetic field to oscillate SPIO. The results of this study showed that these cross-linking disulfiram/SPIO/ALG MPs are potential drug carriers for the treatment of ovarian cancer.

## 1. Introduction

Epithelial ovarian cancer (EOC) is typically diagnosed at advanced stages, and is associated with a high relapse rate and is the leading cause of death because the symptoms are usually nonspecific until they have metastasized [1,2,3,4,5]. The treatment of ovarian cancer requires intensive surgical intervention and complex chemotherapies [6]. Although responsive to cytoreductive surgery and chemotherapy initially, recurrence with intraperitoneal metastasis and chemoresistance is common [7,8]. If left untreated, the tumor can spread to other parts of the body. This is called metastatic ovarian cancer. The earliest symptoms of ovarian cancer are vague and easy to dismiss. As a result, only 20% of ovarian cancers are detected at an early stage [1]. The late identification of ovarian cancer gives it more time to develop, and makes it easier to become metastatic ovarian cancer. Metastatic ovarian cancer is an advanced-stage malignancy that has spread from the cells in the ovaries to distant areas of the body. The abdomen peritoneum, lymph nodes or surrounding organs are the most commonly seen metastatic-targeted sites. Currently, chemotherapy is the most effective way to treat metastatic cancer, and the administration route is normally via intravenous injection to ensure systematic circulation. The disadvantage of this treatment is that it raises lots of side effects due to the nonselective affection of cells including normal tissue cells. Thus, patients are frequently suffering severe side effects and losing quality of life after treatment. This nonselective treatment inspires us to develop a more effective, selective, and more targeted way of therapy toward cancerous cells. Considering the efficiency of drug delivery and avoidance of the systemic circulation, a direct delivery of the drug to the affected site and a combined therapy consisting of lower dosage of chemotherapeutic reagent with other complementary medicine technology occurred to us for developing a new way for treating those peritoneal carcinomatosis, which is a frequently seen metastasis cancer from ovarian cancer and is easily spread in the abdomen cavity without obvious symptoms. To conquer this troublesome primary cancer and its accompanying metastasis, several treatments were already developed to combine cytoreduction surgery with hyperthermic chemotherapy, such as hyperthermic intraperitoneal chemotherapy (HIPEC) [9]. In addition, other targeted therapies such as using antiangiogenic agents, poly (ADP-ribose) polymerase inhibitors, or hyperthermia treatment given by superparamagnetic iron oxide nanoparticles (SPIO) are another group of emerging therapeutic modalities [10,11]. Poly (ADP-ribose) polymerase inhibitors exhibited mechanisms on pleiotropic cellular functions ranging from the maintenance of genomic stability and chromatin remodeling to the regulation of cell death, thereby rendering PARP homologues promising targets in cancer therapy. SPIO nanoparticles showed magnetic hyperthermia function once an external magnetic field was provided to induce magnetic oscillation and friction. It is particularly noteworthy that some of the old drugs, such as disulfiram for treating chronic alcoholism, were repurposed to discover new potential uses, such as a novel therapeutic approach against gynecological neoplasm [12]. Based on Y.A. Rezk et al.’s research work, they found that disulfiram induced apoptotic cell death, and the cytotoxicity of disulfiram was comparable to that of cisplatin (Cisplatin IC50: ~3 µM). Although ovarian cancer cell lines are most sensitive to disulfiram, the normal human ovarian surface epithelial cells (HOSE) were modestly sensitive to disulfiram, which means the surrounding cells of normal tissue around the primary cancer or metastatic cells are potentially affected by the disulfiram treatment while retarding the growth of cancerous cells. This current problem of using disulfiram inspires us to develop a new strategy for drug delivery against ovarian cancer cells without harming the normal tissue cells.

In our previous study [13], we determined that when we strike a balance between the stirring rate (related to the shear force) and cross-linking degree (related to the hardness of the particle), the shape control of the resultant microparticles (MPs) could be reached and produced under the assistance of the electrospray technique. As a major difference from the common electrospray technique [14], our electrospray setup was in conjunction with an in situ cross-linking process. The shape of the particle can be varied from a peanut shape to a disc shape, with up to 95.43% ± 1.84% monodispersed shape distribution. Importantly, we found that the disc-shaped microparticles tended to settle on the flat surface and guaranteed that the drug-delivery vehicles could greatly adhere and bind to the targeted site. Moreover, our preliminary in vitro cell model assays revealed that these disc-shaped microparticles demonstrated an encapsulation efficiency of 98% toward the disulfiram drug, and a cell toxicity effect of reduction of 20–40% growth toward the ovarian cancer cell lines SKOV-3 and CP70 was achieved by using the disc-shaped microparticle, whose effect was similar to that of three times of the free-form dosage in treatment groups. All of these positive responses in cell model tests inspired us to design and initiate a xenograft ovarian cancer-bearing animal model for testing a combined therapy against ovarian cancer in this study. This novel combined therapy consisted of a novel chemotherapeutic reagent, i.e., disulfiram, and a hyperthermia trigger, i.e., iron oxide nanoparticles. These two therapies were installed in a disc-shaped microparticle, which signifies that the disc-shaped microparticles simultaneously provide combined therapies once the disc-shaped microparticle attaches to the affected area. As the disc-shaped microparticle suspension was directly injected into the primary cancer site and then filled up the surrounding tissue, when an alternating magnetic field was applied, the microparticles heated up the cancerous tissue and also stimulated the release of the drug. We found that a dramatic reduction (approximately 66% reduction as compared to free-form disulfiram) in disulfiram dosage used was observed in the combined-therapy group to achieve the same efficacy when only full dosage of disulfiram was given. According to the obtained temperature record after applying the alternating magnetic field, we believe that a temperature raise to 42 °C helped with accelerating the cancerous cell apoptosis.

## 2. Materials and Method

### 2.1. Materials

Alginate was obtained from Sigma-Aldrich (cat. no. A1112-100G, low viscosity, 4–12 cP, 1% in H_2_O (25 °C), mannuronate and gluronate ratio (M/G ratio), St. Louis, MO, USA) falling in the range of 6.07–6.18 ± 0.04–0.06, estimated from the cited reference) [15]. Calcium chloride anhydrous (99%, J.T. Baker, NJ, USA), Optiray^®^ 350 (Guerbet, ioversol injection 74%, Villepinte, France), Super-Paramagnetic Ionic Oxide (SPIO; USPIO-118, 10 mL, TANBead, Taiwan Advanced Nanotech Inc, Taoyuan, Taiwan), disulfiram (PHR1690-1G, Sigma-Aldrich), and phosphate buffer saline (PBS, UR-PBS001-5L, UniRegion Bio-Tech, Hsinchu, Taiwan) were all purchased from the manufacturer without further purification.

### 2.2. Preparation of Peanut, Quasi-Peanut, and Disc-Shaped Microparticles

For each run of the synthesis, 0.1 g of alginate powder, 55.2 mg of disulfiram tablet (containing 40 mg effective disulfiram ingredient), and 0.25 mL of the SPIO suspension was dissolved in 1 mL of deionized water and 1 mL of the as-received ioversol solution. This mixture was kept under 800 rpm, stirring till all substances were homogeneously dissolved. Subsequently, 1 g, 2 g, 4 g, or 8 g of calcium chloride aqueous solution was dissolved in 50 mL of deionized water to generate a series of calcium chloride cross-linking solutions (2 wt%, 4 wt%, 8 wt%, and 16 wt%, respectively) for collecting electrosprayed alginate particles. An electrospray (ES) system was established as described in a previous study of ours. [13] Firstly, we used a syringe pump system (NE-300 Just Infusion; New Era Pump Systems, Farmingdale, NY, USA) to inject the stock solution for the electrospray process. A 20-gauge flat-tipped needle was used as the capillary tube in this study. A 15 ± 2 kV positive voltage was given to the spray needle using a direct-current high-voltage power supply (Bertan Model 205B-20R; Spellman High Voltage Electronics, Hauppauge, NY, USA) to build an electrical field between the capillary needle and the electrically grounded collection substrate. The collection substrate in this setup was a 2–16 wt% calcium chloride aqueous solution and the stirring rate was kept between 0–1000 rpm, depending on the intended shape control needs. The capillary nozzle was placed at a working distance of approximately 2 cm from the collection substrate. The ES modes of the system were monitored by viewing the liquid meniscus at the exit of the capillary nozzle. In addition, the meniscus was illuminated with diffuse light from an optical cable light, and its droplet shape was observed using a microscopic system comprising a microscopic lens module (model: DFK22AUC03, The IMAGINGSOURCE, Bremen, Germany), digital camera system (model: STC-620PWT, Sentech, Carrollton, TX, USA), and a liquid crystal display panel. Finally, the obtained MPs were collected by the collection solution. Then, a drop of the produced suspension was dropped onto the glass slide and the specimen could be quickly visualized and the shape of the produced microparticles was determined. The desired shape of the microparticles was produced by using parameter pairs that could help with controlling the product morphology to fall in disc shape. These disc-shaped microparticles were used for subsequent characterization and assaying. The amount of total encapsulated disulfiram was next determined through spectrophotometry on a SPECTROstar Nano instrument (BMG LABTECH, Ortenberg, Germany).

### 2.3. In Vitro Cell Model Studies of Disulfiram-Encapsulating Disc-Shaped Microparticles against Ovarian Cancer Cell Lines

We performed an MTT assay (tetrazolium salt 3-(4,5-dimethylthiazol-2-yl)-2,5-diphenyltetrazolium bromide) to evaluate the cell viability of SKOV-3 and CP70 after they had been treated with the disc-shaped microparticles suspension or the microparticle suspension plus applied alternating magnetic field. The alternating magnetic field was supplied by an induction generator (Power Cube 64/900, CEIA, Arezzo, Italy). In a typical procedure, a density of 10^3^–10^4^ cells/100 μL (100 μL/well) in a serum-containing RPMI-1640 medium suspension of SKOV-3 or CP70 cells were seeded onto a 96-well plate for 12 h at 37 °C in a 5% CO_2_ atmosphere environment. After 12 h of culture period, the medium was pipetted away, and 100–300 μM of disulfiram-encapsulating disc-shaped microparticle suspensions were added to each well for drug treatment for 24 h or drug treatment plus 15 min of magnetic field applied. After an extra 72 h of incubation period at 37 °C in a 5% CO_2_ atmosphere environment, the supernatant was taken away from each well and the cells were rinsed twice with a fresh 200 μL of 1 × PBS solution. Finally, 100 μL of the MTT reagent was pipetted to each well and the plate was subsequently incubated for 2 h until purple precipitate was observed. After 2 h, all supernatants were decanted and replaced with a fresh 200 μL of dimethyl sulfoxide solvent to dissolve the purple crystal. The cell plate was then capped and placed in the dark for 10 min at 37 °C in a 5% CO_2_ atmosphere environment. Ultraviolet-visible (UV−vis) spectrophotometer was used to measure the optical density (OD) of the dimethyl sulfoxide extract solution at 570 nm. Subsequently, cell viability was calculated as the ratio of the recorded absorbance values by using the following equation:

Cell viability = absorbance_570_ of the drug-treated group/absorbance_570_ of the medium-only group × 100%

Culture was incubated at 37 °C incubator equipped with humidified atmosphere with 5% CO_2_ gas.

### 2.4. In Vitro Drug Release of the Disulfiram-Encapsulating Disc-Shaped Microparticles

A fixed amount of the disulfiram-encapsulating disc-shaped microparticles was suspended in 3 mL simulated body fluid (SBF). This microparticle suspension was then shaken at 100 rpm and maintained at 37 °C with a hot plate equipped with a magnetic stirrer. At a predetermined time interval (1, 2, 3, 4, 5, 6, 9, 12, 24, 48, 72 h), the entire SBF solution in the beaker was removed and replaced with 3 mL of fresh SBF. The disulfiram concentration in the used SBF extract solution was subsequently measured via absorbance at 285 nm (the maximum ultraviolet absorption wavelength of disulfiram). Finally, the cumulative percentage of drug release at a specific time interval was calculated based on the calibration line established and is shown in the following figures.

### 2.5. In Vivo Animal Model Studies of Disulfiram-Encapsulating Disc-Shaped Microparticles against Ovarian Cancer Cell Lines

Female nude mice (strain: CAnN.Cg-Foxn1nu/CrlNarl, 6 weeks, 17–20.7 g) were purchased from the National Laboratory Animal Center (Taipei, Taiwan). Experiments were conducted in accordance with institutional guidelines and were approved by the National Defense Medical Center’s Institutional Animal Care and Use Committee under certificate no. IACUC-20-333 (13 November 2020). The mice were grouped and housed (*n* = 3 per cage) in polypropylene cages, with free access to food and water. The vivarium was maintained on a 12 h light: 12 h dark cycle, with a room temperature of 22 ± 1 °C and relative humidity level of 50 ± 5%, with food (laboratory rodent diet, labdiet 5001, Labdiet, St. Louis, MO, USA) and water ad libitum. All studies were in compliance with the rules set forth in the Guide for the Care and Use of Laboratory Animals.

Female nude mice at 6 weeks of age were divided into CP70 and SKOV3 tumor groups, receiving 4 kinds of drug delivery: (1) tumor-bearing mice receiving phosphate-buffer saline (negative control), (2) tumor-bearing mice receiving cisplatin (positive control), (3) tumor-bearing mice receiving an intraperitoneal injection of 1 mg/mouse dosage of disulfiram-encapsulating disc-shaped microparticle suspension, and (4) tumor-bearing mice receiving an intraperitoneal injection of 1 mg/mouse dosage of disulfiram-encapsulating disc-shaped microparticle suspension plus 10 min of alternating magnetic field illumination. CP70 or SKOV3 tumor cells (4.3 × 10^5^) in a volume of 500 μL were injected via the intraperitoneal route into the abdomen cavity of nude mice. Following inoculation of tumor cells or PBS, 3–4 weeks was needed to obviously see the successful induction of tumor growth and generation of ascites. In this tumor inoculation period, tumor mass, food intake, and tumor size were measured three times weekly. Tumor growth evidence was assessed by abdominal palpation, whereas drug treatment started from day 21–28 after inoculation when a palpable tumor was felt. A total of 15 doses were given to each group and all doses were injected via an intraperitoneal injection. After reaching the endpoint, all mice were sacrificed and their tumor, liver, and spleen organs were harvested. Tumor growth was assessed by the measurement of two bisecting diameters in each tumor using a ruler, and the tumor volume was calculated using the following equation:

tumor volume (mm^3^) = 1/2 × width × length^2^

### 2.6. Histological Sample Preparation and Analysis

In the previous in vivo animal study, the primary tumor, liver, and spleen organs were harvested after sacrifice. For the histological analyses, all tissues including spleen, pancreas, liver, kidney, ovarian, and tumor that were taken from the mice were postfixed in 4% formaldehyde overnight in a 4 °C freezer. Subsequently, all specimens were dehydrated in gradient ethanol solution (from 70% to 100%). The posttreated specimens were embedded in paraffin and sectioned at a 5 μm thickness, then mounted on a glass slide for histological analyses. For hematoxylin–eosin staining, sections were stained in hematoxylin dye for 5 min, and then in eosin dye for 5 min according to the protocol given by the manufacturer of the staining kit. All slides were inspected with a Leica DMIL LED inverted microscope (Leica, Wetzlar, Germany).

### 2.7. Statistical Analysis

Statistical analysis was carried out employing Wilcoxon statistics, one-way ANOVA, and a Student’s *t*-test using SPSS software (SPSS, Chicago, IL, USA) to assess the differences between the experimental groups and the positive control group. Statistical results were considered significant when the *p*-value < 0.05 (*).

## 3. Results

**Figure 1:**
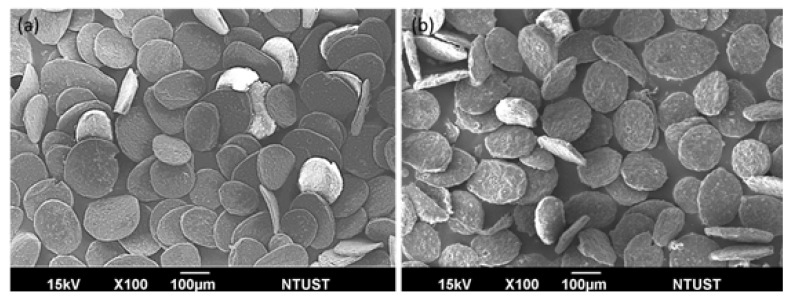
Scanning electron microscopy (SEM) images of disc-shaped (**a**) ALG MPs, and (**b**) disul/SPIO/ALG MPs.

**Figure 2:**
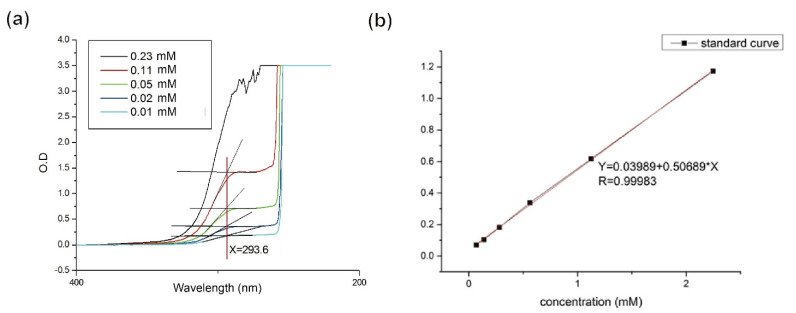
Ultraviolet-visible (UV–vis) spectrum: (**a**) UV–vis spectrum acquired from a series of predetermined concentration of disulfiram solution, and (**b**) disulfiram drug calibration line established according to the maximum optical density obtained at 293.6 nm indicated in Figure 2a.

In our previous study [13], several morphology-related parameters were investigated and a pair of parameters was found to strongly dominate the generation of particle morphology, i.e., the rpm of the stirring rate and the cross-linking degree. Among various shapes of alginate microparticle generation, disc-shaped particles are the most interesting, as only this shape can attach well to the flat surface. For example, the targeted application in this study was aiming at providing combined therapy on ovarian cancer treatment by a spray administration route. Figure 1 shows the scanning electron microscopy (SEM) images of alginate microparticles with disulfiram–SPIO–coencapsulation (disul/SPIO/ALG MPs) with particle size falling in 251.5 ± 40.2 μm. As can be seen in the images, Figure 1a shows the blank SPIO/ALG MPs with a smooth and flat surface. Figure 1b shows the MPs with disulfiram incorporation (disul/SPIO/ALG MPs). It is worth noting that the surface smoothness became rough and uneven; however, the addition of disulfiram did not alter the formation of disc-shape disul/SPIO/ALG MPs. For the encapsulated SPIO particle, their encapsulation and distribution in the MPs has been well investigated in our previous study [13] and the physical and chemical properties of the SPIO is already well investigated [16]. This indicates that the plausible mechanism we found in the generation of blank SPIO/ALG MPs as follows is still a dominant factor: the shearing effect is still needed to strike a balance with the hardness (i.e., cross-linking degree) of the particle to work synergistically to produce the disc-shaped microparticles. By controlling two factors, namely the stirring rate and calcium chloride concentration, the particle shape could be controlled (spherical-, peanut-, and disc-shaped particles). However, considering future in vivo studies for combined therapy on ovarian cancer by the spray administration route, disc-shaped disul/SPIO/ALG MPs were chosen as the candidates for the following in vitro and in vivo studies. To understand the drug-encapsulation efficiency and the quantitative determination of the drug amount, citric acid was used to chelate the calcium ion (thus generating calcium citrate) in the cross-linking disul/SPIO/ALG MPs and then break down the particle structure. Figure 2a shows that when 2% of citric acid was adopted, the maximum concentration of disul, approximately 2.24 mM, could be detected and an UV absorption peak was observed at 285 nm (as the red solid line indicates). Based on this optical absorption result, a calibration line of the disul drug was established for quantitative purposes (Figure 2b). A whole batch of freshly prepared disc-shaped disul/SPIO/ALG MPs were subject to the encapsulation efficiency determination and 98.89% efficiency was obtained.

$$\mathrm{Encapsulation}\;\mathrm{Efficiency}\left(\%\right)=\frac{\mathrm{Weight}\;\mathrm{of}\;\mathrm{the}\;\mathrm{drug}\;\mathrm{in}\;\mathrm{microparticles}}{\mathrm{Weight}\;\mathrm{of}\;\mathrm{the}\;\mathrm{feeding}\;\mathrm{drugs}}\times 100$$

**Figure 3:**
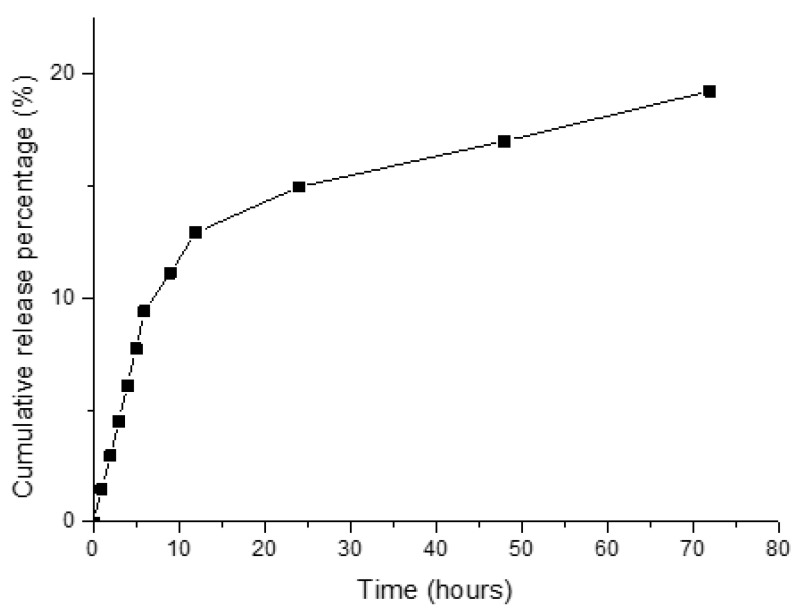
Drug release profile acquired from the cross-linking disul/SPIO/ALG MPs.

Figure 3 illustrates the release profile acquired from the cross-linking disul/SPIO/ALG MPs at various time points in a simulated body fluid (SBF) solution. Obviously, as the disul drug was encapsulated inside the alginate matrix, the release rate was relatively slow and reached only 20% of drug release after 72 h of the test period. Although the release rate and amount was low, this can greatly reduce the side effect caused by aldehyde dehydrogenase (ALDH) inhibition in the liver. The plausible release mechanism was that the cross-link divalent calcium ions were partially replaced and reacted with HPO_4_^-^ ions in the SBF solution. This generated the loss of the cross-linking structure of the alginate matrix.

**Figure 4:**
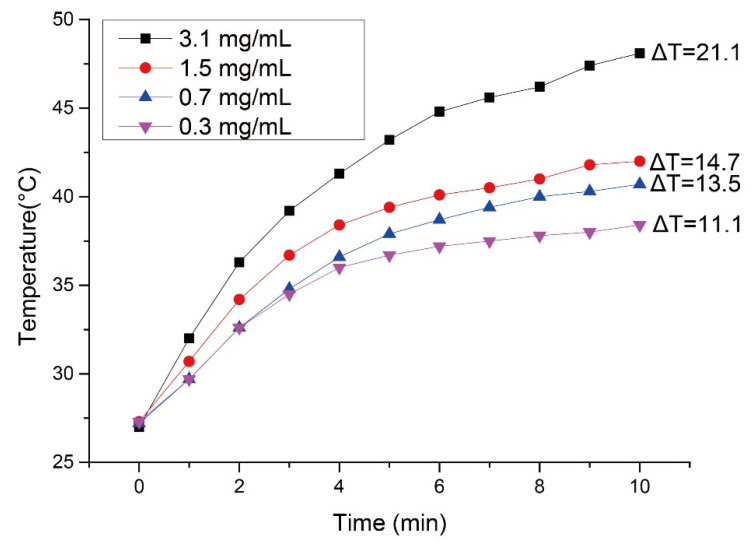
A series of suspensions with different SPIO concentrations were tested under the same strength of high frequency induction heating generator (Power Cube 64/900, CEIA) and the temperature-elevated curve was recorded versus time.

To observe the optimum dosage of SPIO encapsulation inside microparticles for sensitizing the alternating magnetic field, a series of suspensions with different SPIO concentrations were tested under the same strength of high frequency induction heating generator (Power Cube 64/900, CEIA, Arezzo, Italy) and the temperature-elevated curve was recorded versus time. (Figure 4). Obviously, as the SPIO concentration was below 1.5 mg/mL, the temperature of the aliquot was not able to reach above 40 °C, which is generally considered an ideal hyperthermia temperature for killing cancerous cells. Once the concentration of the SPIO was raised to 3.1 mg/mL, the temperature of the suspension abruptly raised to approximately 47.5 °C in just 15 min of hyperthermia treatment. This high temperature might cause the denatured state of the protein in normal cells. Therefore, a 1.5 mg/mL dosage was chosen to strike a balance between hyperthermia efficacy and treatment safety.

**Figure 5:**
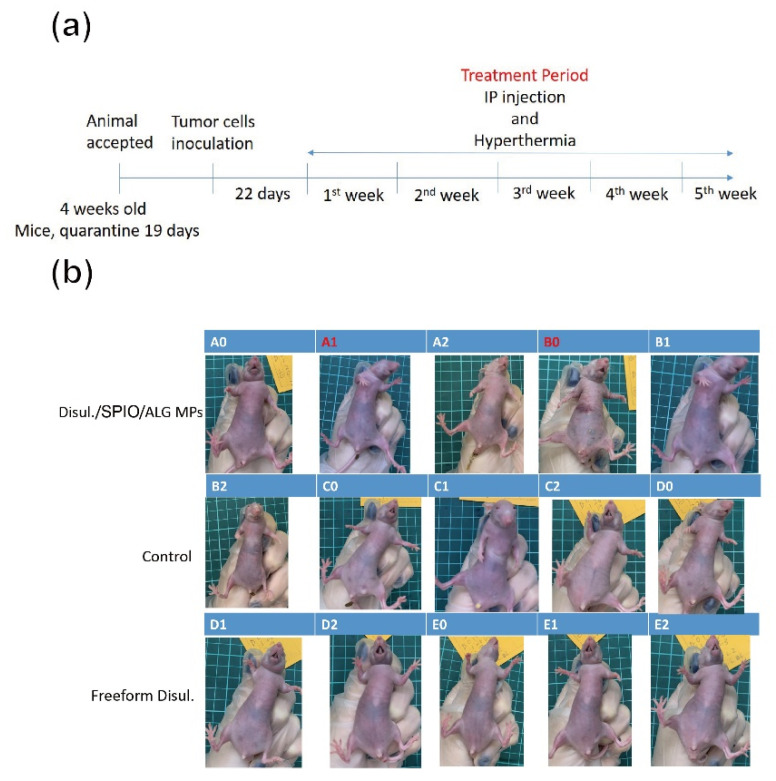
(**a**) Timeline for an in vivo xenograft tumor-bearing mice model animal test, and (**b**) optical images recorded for all groups of mice after receiving a complete cycle of treatment.

**Figure 6:**
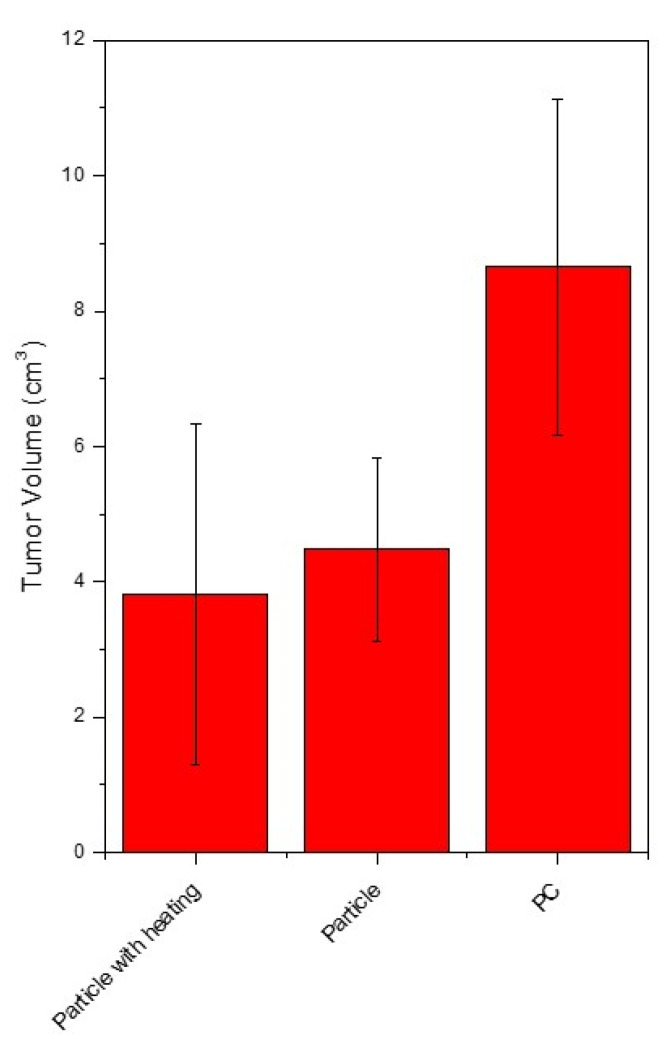
Histological results for all groups of tumor volume estimation with/without combined therapy. Particle- and particle with heating-treated group both show *p*-value < 0.05.

Figure 5a depicts the timeline for an in vivo xenograft tumor-bearing mice model animal test. After the immune deficient mice were successfully induced with tumor growth in the abdomen cavity, a consecutive treatment of disc-shaped disul/SPIO/ALG MPs via IP injection and then application of alternating magnetic field was provided for each mouse every week. This treatment combined the chemotherapy and hyperthermia together. The hyperthermia temperature was controlled at around 41–45 °C. The endpoint of the mice was set at 7 weeks after the tumor was successfully induced. Figure 5b shows the outlook for all groups of mice after 7 weeks of combined therapy treatment. As the tumor was embedded for growth inside the abdomen cavity, no apparent abdominal distension was observed, although tumor growth was out of control, as shown in Figure 5b (case C1, C2 and D0). On the contrary, for the disul/SPIO/ALG MPs-treated group, it is worthy of note that the body of the mice is slender compared to those in the control group. This evidence indicated that the tumor growth in the disul/SPIO/ALG MPs-treated group of mice might be well controlled after treatment except that disseminated intravascular coagulation was seen in this group (see a typical case B0 in Figure 5b). After sacrificing of all mice, primary tumors at the ovarian organ were harvested and submitted for tumor volume estimation and pathological analysis. Figure 6 shows the results for all groups of tumor volume estimation with/without treatment. Control group mice were provided a cisplatin injection treatment, which is the currently used clinical chemotherapy. An obvious suppression of tumor growth was observed in both freeform disul and disul/SPIO/ALG MPs-treated groups, which show a 48.2% and 55.9% tumor volume reduction, respectively, as compared to the control group.

**Figure 7:**
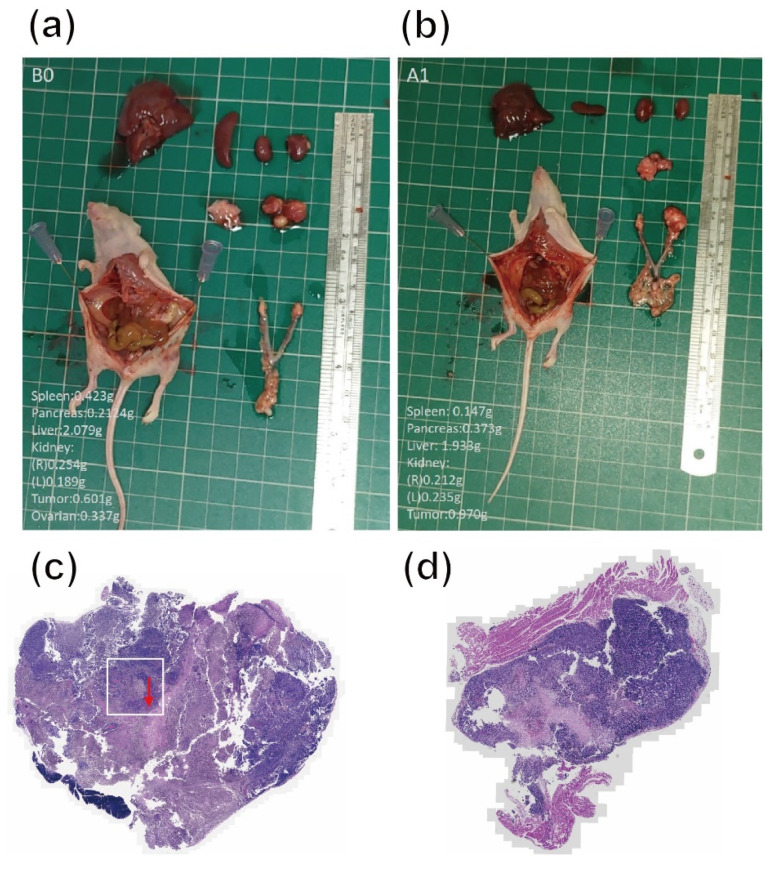
Representative images of open surgery for the sacrificed mice in (**a**,**b**) disul/SPIO/ALG MPs-treated groups, which indicates that the tumor growth was greatly reduced: (**a**) tumor in B0 mice even completely disappeared. However, obvious hepatomegaly was observed (see large liver tissue volume, especially in B0 mouse case), and (**b**) tumor growth was only found on single side of the ovarian in A1 mouse. (**c**,**d**) show the H–E staining of the tumor tissue harvested from the control group and disul/SPIO/ALG MPs-treated group, respectively. The red arrowhead indicates that there are many angiogenesis blood vessels in the square area.

Furthermore, Figure 7a,b shows the representative images of open surgery for the sacrificed mice in disul/SPIO/ALG MPs-treated groups. Although an adverse effect of hepatomegaly was observed for all surviving mice, the ovarian cancer occurred at the primary site indeed disappeared or became less infiltrating (see indicated red arrowheads). This result proves that our hypothesis made in our previous study in vitro, i.e., to strike a balance between the beneficial effect and side effects of the drug, 100 μM disulfiram-encapsulated disc-shaped SPIO-encapsulated alginate microparticles were considered the optimum formulation in the cell model, still works in an in vivo xenograft animal model by IP injection with an externally magnetic field applied. Figure 7c,d shows the H–E staining of the tumor tissue harvested from the control group and disul/SPIO/ALG MPs-treated group, respectively. It is clear to see that the tumor volume of the disul/SPIO/ALG MPs-treated group is smaller than that found in the control group. Moreover, it is worth noting that the red arrowhead indicated in Figure 7c shows that there are many angiogenesis blood vessels in the square area.

## 4. Discussion

In general, a reliable protocol was developed in this study for the shape-controlled synthesis of novel multicomponent-encapsulated alginate MPs, which can simultaneously provide chemotherapy and hyperthermia treatment to the ovarian cancer site. One of the main purposes of this combined therapy development is to conquer possible metastasis cancer cell implantation in the surgical procedure and remove the drawback of the currently used therapy, i.e., HIPEC. As HIPEC therapy needs to be given to the patient right after the primary surgery with the abdominal cavity open, a chemotherapy fluid heated to a temperature greater than the normal body temperature is injected via the intraperitoneal (ip.) route and continuously circulates chemotherapeutic agents throughout the peritoneal cavity, for 1.5–2 h. This tedious procedure makes the infection risk for the patient rise, and the treatment can only be given before the closure of the wound. On the contrary, this newly developed combined therapy can be provided by sprays of the MPs to adhere to the internal lining of the abdominal cavity which carry a chemotherapy reagent and magnetic SPIO at the same time. The chemotherapy reagent can slow release and the hyperthermia treatment can be given repeats even after the wound closure of the abdomen by externally providing an alternating magnetic field. In conjunction with hyperthermia, the cancer cell response to the chemotherapy reagent improves and low dosages can achieve satisfactory efficacy. Moreover, use of acute poison chemotherapy reagents can be avoided to reduce the notorious side effects expected and the sacrificing of patient’s life quality. Although disulfiram has an off-label use of inhibiting the growth of ovarian cancer cells, this new combined formulation was designed to be given by direct spray at the affected area, which could greatly reduce the dosage used and side effects such as vomiting and nausea. An in vivo study indeed shows great tumor volume reduction and suppression (up to 55.9 ± 34%), respectively, as compared to the control group, which shows the potential and future applications of this old drug on ovarian cancer therapy.

## 5. Conclusions

In conclusion, as can be seen in our in vivo study, the ip. injection of cancerous cells only induced primary tumors after receiving our combined therapy. Moreover, the primary tumor seems to be completely suppressed of growth or occurs only on one side of the ovaries. This indicates that our combined therapy not only retards the metastasis but also reduces the primary tumor growth simultaneously. This treatment can be applied several times until reduction of the tumor volume is observed. In comparison to HIPEC, this method of treatment prevents the risk of infection and anaesthesia risk during long-time open-wound surgery on the abdominal cavity. However, side effects such as hepatomegaly and disseminated intravascular coagulation were increasing after receiving this combined therapy and possibly resulted from the imprecise temperature during hyperthermia treatment period. This phenomenon reveals that when people intend to use this combined therapy, a precise dosage of disul/SPIO/ALG MPs and magnetic field given are in strong need.

## Data Availability

Data available on request due to restrictions e.g., privacy or ethical. The data presented in this study are available on request from the corresponding author. The data are not publicly available due to future patent application.
